# Effects of *Caenorhabditis elegans sgk-1* mutations on lifespan, stress resistance, and DAF-16/FoxO regulation

**DOI:** 10.1111/acel.12120

**Published:** 2013-07-19

**Authors:** Albert Tzong-Yang Chen, Chunfang Guo, Kathleen J Dumas, Kaveh Ashrafi, Patrick J Hu

**Affiliations:** 1 Life Sciences Institute, University of MichiganAnn Arbor, Michigan; 2 Department of Physiology, University of CaliforniaSan Francisco, California; 3 Departments of Internal Medicine and Cell and Developmental Biology, University of Michigan Medical SchoolAnn Arbor, Michigan

**Keywords:** aging, *C. elegans*, FoxO, insulin-like growth factor signaling, lifespan, Sgk

## Abstract

The AGC family serine–threonine kinases Akt and Sgk are similar in primary amino acid sequence and *in vitro* substrate specificity, and both kinases are thought to directly phosphorylate and inhibit FoxO transcription factors. In the nematode *Caenorhabditis elegans*, it is well established that AKT-1 controls dauer arrest and lifespan by regulating the subcellular localization of the FoxO transcription factor DAF-16. SGK-1 is thought to act similarly to AKT-1 in lifespan control by phosphorylating and inhibiting the nuclear translocation of DAF-16/FoxO. Using *sgk-1* null and gain-of-function mutants, we now provide multiple lines of evidence indicating that AKT-1 and SGK-1 influence *C. elegans* lifespan, stress resistance, and DAF-16/FoxO activity in fundamentally different ways. Whereas AKT-1 shortens lifespan, SGK-1 promotes longevity in a DAF-16-/FoxO-dependent manner. In contrast to AKT-1, which reduces resistance to multiple stresses, SGK-1 promotes resistance to oxidative stress and ultraviolet radiation but inhibits thermotolerance. Analysis of several DAF-16/FoxO target genes that are repressed by AKT-1 reveals that SGK-1 represses a subset of these genes while having little influence on the expression of others. Accordingly, unlike AKT-1, which promotes the cytoplasmic sequestration of DAF-16/FoxO, SGK-1 does not influence DAF-16/FoxO subcellular localization. Thus, in spite of their similar *in vitro* substrate specificities, Akt and Sgk influence longevity, stress resistance, and FoxO activity through distinct mechanisms *in vivo*. Our findings highlight the need for a re-evaluation of current paradigms of FoxO regulation by Sgk.

## Introduction

Akt/protein kinase B (PKB) and Sgk are two highly related members of the AGC family of serine–threonine kinases that act in cellular signaling pathways to modulate survival, growth, proliferation, metabolism, and other processes (Pearce *et al*., 2010). Akt/PKB has evolutionarily conserved functions in the control of development, growth, metabolism, cell survival, and longevity, and dysregulation of Akt/PKB contributes to the pathogenesis of common human diseases such as cancer and type 2 diabetes (Franke, 2008).

The mechanism of Akt/PKB activation is well established. In response to growth factors, Akt/PKB is activated in a phosphoinositide 3-kinase (PI3K)-dependent manner by phosphorylation at two critical regulatory sites: T308 within its kinase domain and S473 within a C-terminal hydrophobic motif (Alessi *et al*., 1996a). The 3-phosphoinositide-dependent kinase PDK1 phosphorylates Akt/PKB at T308 (Alessi *et al*., 1997; Stephens *et al*., 1998), and members of the PI3K-related kinase (PIKK) family such as TOR complex 2 phosphorylate Akt/PKB at S473 (Feng *et al*., 2004; Sarbassov *et al*., 2005; Viniegra *et al*., 2005).

Activated Akt/PKB phosphorylates several substrates *in vivo* at sites that lie within RxRxxS/T motifs (Alessi *et al*., 1996b; Manning & Cantley, 2007). Among these substrates are the FoxO family of transcription factors that control development, metabolism, growth, and aging (Accili & Arden, 2004). Akt/PKB-dependent phosphorylation of FoxO at three conserved RxRxxS/T motifs inhibits FoxO activity by promoting its export from the nucleus and sequestration in the cytoplasm (Brunet *et al*., 1999). FoxO is a critical substrate of Akt/PKB *in vivo*, as its disinhibition in mice with reduced hepatic Akt/PKB signaling impairs metabolic homeostasis (Dong *et al*., 2008), and a null mutation in *daf-16*, which encodes the sole FoxO transcription factor in the nematode *Caenorhabditis elegans*, suppresses the dauer-constitutive and lifespan extension phenotypes of animals with reduced Akt/PKB activity (Paradis & Ruvkun, 1998; Kwon *et al*., 2010). Thus, Akt/PKB has an evolutionarily conserved function as a direct inhibitor of FoxO transcription factors.

The serum- and glucocorticoid-regulated kinase gene *sgk* encodes a serine–threonine kinase highly homologous to Akt/PKB that was first identified as a gene whose transcription is induced acutely by serum and glucocorticoids in a rat mammary tumor cell line (Webster *et al*., 1993). Like Akt/PKB activation, Sgk activation by growth factors is PI3K dependent and involves the phosphorylation of a site in the kinase domain (T256) by PDK1 (Kobayashi & Cohen, 1999; Park *et al*., 1999) and a C-terminal site within a hydrophobic motif by TOR complex 2 (Garcia-Martinez & Alessi, 2008). Furthermore, Sgk also phosphorylates sites that lie within RxRxxS/T motifs (Kobayashi & Cohen, 1999). In spite of these similarities, some Akt/PKB substrates are poor substrates for Sgk *in vitro* and *vice versa* (Murray *et al*., 2004a,b2004b); this is likely due at least in part to amino acids in the vicinity of the phosphoacceptor residue that confer substrate specificity (Murray *et al*., 2005). The distinct substrate specificities of Akt/PKB and Sgk are reflected in the observation that although both mammalian Akt/PKB and Sgk can promote the phosphorylation of the FoxO3 transcription factor in cultured cells at sites within all three conserved RxRxxS/T motifs, they do so with distinct efficiencies within each motif (Brunet *et al*., 2001).

In mammalian cell culture, Sgk inhibits FoxO3 activity (Liu *et al*., 2000; Brunet *et al*., 2001), and in *C. elegans,* SGK-1 is thought to limit lifespan by inhibiting DAF-16/FoxO activity (Hertweck *et al*., 2004). Taken together with the known role of Akt/PKB in FoxO regulation, these studies have established a paradigm whereby Akt/PKB and Sgk are thought to act via similar mechanisms to inhibit FoxO activity (Fielenbach & Antebi, 2008; Bruhn *et al*., 2010; Pearce *et al*., 2010).

We and others recently reported that in contrast to the lifespan extension phenotype observed after RNAi knockdown of *sgk-1* (Hertweck *et al*., 2004), *sgk-1* null mutations shorten *C. elegans* lifespan (Soukas *et al*., 2009; Alam *et al*., 2010; Kwon *et al*., 2010). This phenotype is the opposite of that observed for *akt-1* null mutations (Soukas *et al*., 2009; Alam *et al*., 2010; Kwon *et al*., 2010) and is inconsistent with prevailing models implicating Sgk as a FoxO inhibitor. In light of these results, we have performed a detailed phenotypic analysis of *sgk-1* null and gain-of-function mutants. Our results indicate that in *C. elegans*, Akt/PKB and Sgk influence lifespan, stress resistance, and FoxO transcription factor activity through distinct mechanisms. These surprising findings call into question current paradigms of FoxO regulation by Sgk and reveal that the interaction between Sgk and FoxO transcription factors may be more complex than previously appreciated.

## Results

### Effects of *sgk-1* mutations on lifespan

**Fig. 1:**
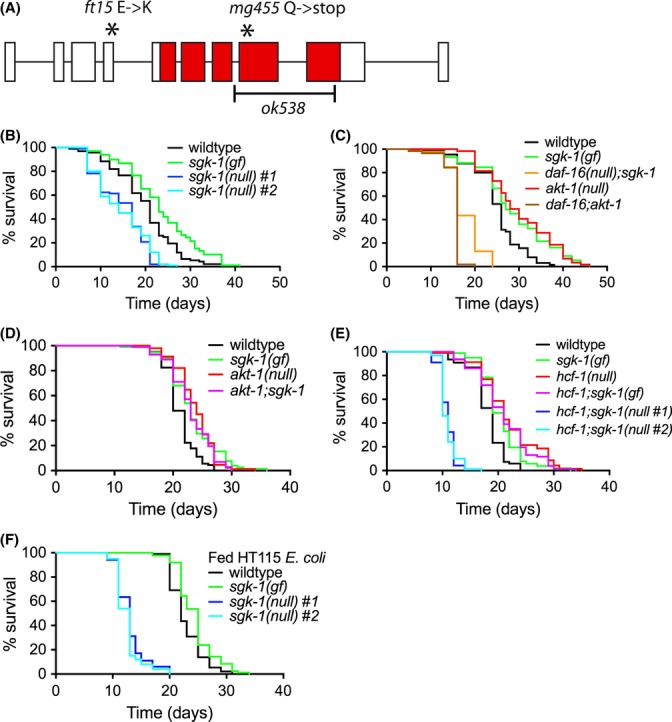
Effects of *sgk-1* mutations on lifespan. (A) Schematic of the *sgk-1* genomic locus (not to scale). Locations of the *ft15* missense gain-of-function*, ok538* deletion*,* and *mg455* nonsense mutations are shown. Exons encoding the kinase domain are colored red. (B) Lifespans of *sgk-1* mutants *ft15* (*gf*), *ok538* (null #1), and *mg455* (null #2). (C) Effect of the *daf-16(mu86)* null mutation on the lifespans of *sgk-1(gf)* animals. (D) Effect of *sgk-1(gf)* on the lifespan of *akt-1(mg306)* null mutant animals. (E) Effect of *sgk-1(gf)* on the lifespan of *hcf-1(pk924)* null mutant animals. Raw data and statistics are presented in Table S1.

We and others have shown that the *sgk-1(mg455)* mutation shortens lifespan (Soukas *et al*., 2009; Alam *et al*., 2010). The *mg455* allele is a nonsense mutation that is predicted to result in truncation of SGK-1 within its kinase domain (Soukas *et al*., 2009); therefore, this is likely to be a null mutation. A third group has shown that the *sgk-1(ok538)* deletion mutation, which is predicted to remove half of the SGK-1 kinase domain and is also probably a null mutation (Hertweck *et al*., 2004), also reduces lifespan (Kwon *et al*., 2010). We confirmed these results by measuring the lifespans of both *sgk-1(ok538)* and *sgk-1(mg455)* null mutants in the same assay (Fig. 1B and Table S1). *sgk-1*(*ok538)* (heretofore referred to as ‘null #1’) and *sgk-1(mg455)* (heretofore referred to as ‘null #2’) each shorten mean lifespan by at least 27.5% and median lifespan by at least 19.0% and 33.3%, respectively (*P* < 0.0001 by the log-rank test). The observation that two outcrossed strains harboring independently isolated *sgk-1* null mutations both have short lifespans compared with wild-type animals strongly suggests that these short lifespan phenotypes are a consequence of reduced SGK-1 activity. These results contrast with the reported lifespan extension induced by *sgk-1* RNAi (Hertweck *et al*., 2004) and are consistent with a model whereby SGK-1 promotes longevity.

One possible explanation for the discrepancy between the lifespans of animals harboring *sgk-1* loss-of-function mutations and animals subjected to *sgk-1* RNAi is that strong loss-of-function mutations could cause developmental abnormalities that shorten adult lifespan by reducing general fitness; such abnormalities can be avoided by initiating RNAi during late larval or early adult stages. To address this possibility, we assayed the lifespans of animals harboring the *sgk-1(ft15)* gain-of-function mutation.

*sgk-1(ft15)* emerged from a genetic screen for suppressors of the developmental delay phenotype of animals harboring a loss-of-function mutation in *lpo-6/rict-1*, which encodes the *C. elegans* ortholog of the TOR complex 2 component Rictor (Jones *et al*., 2009; Soukas *et al*., 2009). *sgk-1(ft15)* suppresses both the developmental delay and small body size phenotypes of *lpo-6/rict-1* loss-of-function mutants, and this suppression is abrogated by *sgk-1* RNAi (Jones *et al*., 2009). Taken together with the observations that *lpo-6/rict-1* and *sgk-1* act in the same genetic pathway (Jones *et al*., 2009; Soukas *et al*., 2009) and mammalian TOR complex 2 activates Sgk by promoting its phosphorylation (Garcia-Martinez & Alessi, 2008), these data strongly suggest that *sgk-1(ft15)* is a gain-of-function allele.

We reasoned that if *sgk-1* null mutants are short-lived because SGK-1 plays a role in promoting longevity, then animals harboring *sgk-1(ft15)* (heretofore referred to as ‘*sgk-1(gf)*’) should live longer than animals with wild-type *sgk-1*. However, if *sgk-1* null mutants are short-lived because they are sick, then *sgk-1(gf)* animals would not be expected to live long. *sgk-1(gf)* animals consistently lived ∼ 15–20% longer than nonsibling wild-type animals (Table S1B). When siblings harboring wild-type *sgk-1* were used as controls, *sgk-1(gf)* animals exhibited a more modest but statistically significant extension in median and mean lifespan in eight of ten experimental trials (Fig. 1B–E and Tables S1A,C). In Fig. 1B, *sgk-1(gf)* increased mean and median lifespan by 17.5% and 9.5%, respectively, compared with wild-type siblings (*P* = 0.0008). This lifespan extension was suppressed by a null mutation in *daf-16/FoxO* (Fig. 1C and Table S1A).

In *C. elegans,* DAF-16/FoxO activity is regulated through at least two mechanisms. Phosphorylation of DAF-16/FoxO by kinases such as AKT-1 inhibits DAF-16/FoxO by promoting its export from the nucleus (Lin *et al*., 2001; Zhang *et al*., 2008; Alam *et al*., 2010; Dumas *et al*., 2010; Kwon *et al*., 2010). Other regulatory proteins such as HCF-1 and EAK-7 inhibit DAF-16/FoxO activity without influencing its subcellular localization (Li *et al*., 2008; Alam *et al*., 2010). To determine whether SGK-1 acts specifically in either of these pathways to promote longevity, we tested the effect of *sgk-1* mutations on the lifespans of *akt-1* and *hcf-1* null mutants. We previously reported that SGK-1 is required for the longevity of *akt-1* mutants (Alam *et al*., 2010). *sgk-1(gf)* did not extend the lifespan of *akt-1(null)* animals (Fig. 1D and Table S1A). Similarly, in *hcf-1(null)* animals, *sgk-1* was required for lifespan extension, but *sgk-1(gf)* did not further increase lifespan (Fig. 1E and Table S1A). Based on these data, whether SGK-1 acts specifically with AKT-1 or HCF-1 to influence lifespan is not clear. It is possible that DAF-16/FoxO activation by SGK-1 is attenuated in backgrounds such as *akt-1(null)* and *hcf-1(null)* in which DAF-16/FoxO is already activated.

As the *Escherichia coli* HB101-derived HT115 strain used in experiments demonstrating that *sgk-1* RNAi extends lifespan (Hertweck *et al*., 2004) differs from the OP50 strain used in our experiments (Fig. 1B–E), we sought to determine the influence of *E. coli* strain differences on the lifespans of *sgk-1* mutants. Therefore, we assayed the lifespans of *sgk-1(null)* and *sgk-1(gf)* mutants grown on HT115. As observed in experiments using OP50 as a food source, *sgk-1(null)* shortened and *sgk-1(gf)* extended lifespan in animals feeding on either HT115 or HB101 (Fig. 1F and Table S1C). Therefore, the prolongevity activity of SGK-1 is not significantly influenced by differences between *E. coli* OP50 and HT115/HB101.

Taken together, these results suggest that in contrast to existing paradigms of FoxO inhibition by Sgk (Brunet *et al*., 2001; Hertweck *et al*., 2004), SGK-1 promotes *C. elegans* longevity in a DAF-16/FoxO-dependent manner.

### Effects of *sgk-1* mutations on dauer arrest

Because DAF-16/FoxO promotes developmental arrest in the dauer larval stage in animals with reduced DAF-2 insulin-like signaling (Vowels & Thomas, 1992; Gottlieb & Ruvkun, 1994), we tested the effect of *sgk-1(null)* and *sgk-1(gf)* on dauer arrest. In agreement with a previous report (Hertweck *et al*., 2004), neither *sgk-1(null)* nor *sgk-1(gf)* had significant effects on dauer arrest at 27°C (Table 1A). Although a significant percentage of *sgk-1(null)* animals arrested during larval development (Table 1A), analysis of these animals using Nomarski microscopy revealed no evidence of dauer alae or pharyngeal constriction (Fig. S3), indicating that these animals were nondauer larvae. In contrast and as previously reported (Hu *et al*., 2006), an *akt-1* null mutation had a strongly penetrant DAF-16/FoxO-dependent dauer-constitutive phenotype under the same assay conditions. Neither *sgk-1(null)* nor *sgk-1(gf)* significantly influenced the dauer-constitutive phenotype of *daf-2(e1368)* (Table 1B). Therefore, SGK-1 does not function in dauer regulation.

**Table 1:** Effects of *sgk-1* mutations on dauer arrest. (A) Dauer formation of *sgk-1* and *akt-1* mutants at 27°C. (B) Effect of *sgk-1* mutations on *daf-2(e1368)* dauer formation at 25°C. Siblings were used in each experiment

	Trial 1		Trial 2		Trial 3		Average (SD)		
					
Genotype	% Dauer	% Non-dauer larvae	% Dauer	% Non-dauer larvae	% Dauer	% Non-dauer larvae	% Dauer	% Non-dauer larvae	*N*
(A) Effects of sgk-1 and akt-1 mutations on dauer formation at 27°C									
Wild-type	0.0	0.0	0.0	0.0	0.0	2.3	0 (0)	0.8 (1.3)	991
akt-1(null)	97.7	0.6	94.5	5.0	89.1	10.9	93.8 (4.3)	5.5 (5.2)	705
daf-16(null);akt-l	0.0	1.8	0.0	9.3	0.0	15.2	0 (0)	8.8 (6.7)	811
sgk-l(gf)	0.0	0.0	0.0	0.0	0.0	10.3	0 (0)	3.4 (5.9)	968
daf-16;sgk-1(gf)	0.0	0.3	0.0	1.9	0.0	23.6	0 (0)	8.6 (13.0)	893
sgk-l(null) #1	0.0	26.7	0.0	52.8	0.0	90.0	0 (0)	56.5 (31.8)	1144
sgk-l(null) #2	0.0	67.5	0.0	46.5	0.0	91.8	0 (0)	68.6 (22.6)	1087

Genotype	Trial 1 % Dauer	Trial 2 % Dauer	Trial 3 % Dauer	Average (SD)	*N*
(B) Effects of sgk-1 mutations on daf-2(e1368) dauer formation at 25°C					
Wild-type	0.0	0.0	0.0	0 (0)	768
sgk-i(gf)	0.0	0.0	0.0	0 (0)	1007
daf-2(e1368)	81.2	90.7	46.8	72.9 (23.1)	882
daf-2;sgk-1(gf)	83.1	86.8	50.3	73.1 (20.1)	596
Wild-type	0.0	0.0	0.0	0 (0)	1018
sgk-1(null) #1	0.0	0.0	0.0	0 (0)	1498
daf-2(e1368)	87.0	90.8	87.1	88.3 (2.2)	1128
daf-2;sgk-1(null) #1	89.8	95.6	94.3	93.2 (3.0)	1016
Wild-type	0.0	0.0	0.0	0 (0)	861
sgk-1(null) #2	0.0	0.0	0.0	0 (0)	1508
daf-2(e1368)	93.5	90.6	83.2	89.1 (5.3)	1064
daf-2;sgk-1(null) #2	93.2	89.5	88.9	90.5 (2.3)	958

### Effects of *sgk-1* mutations on stress resistance

**Fig. 2:**
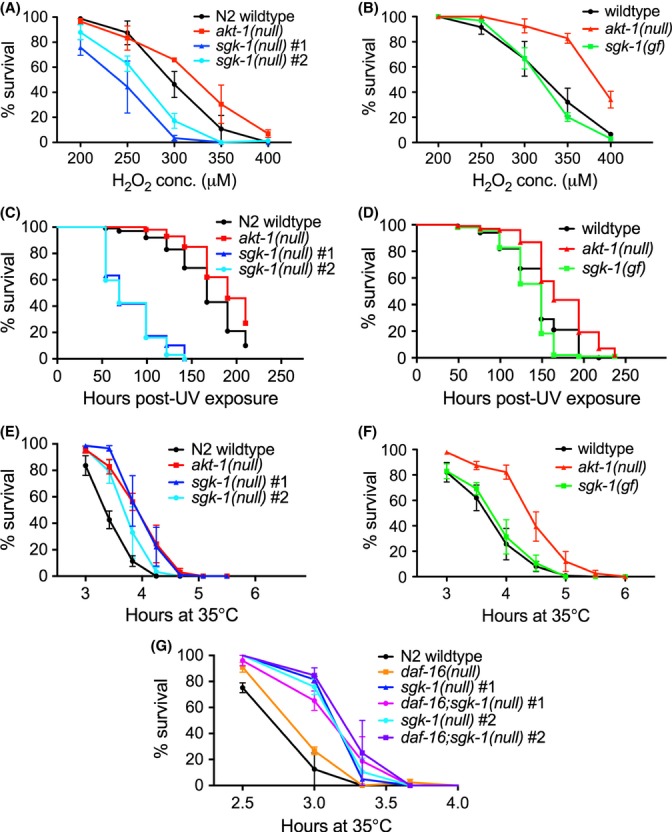
Effects of *sgk-1* mutations on stress resistance. (A–F) Stress resistance assays exposing animals to hydrogen peroxide (A,B), UV radiation (C,D), and heat (E,F). Assays were performed on *sgk-1(ok538)* (null #1), *sgk-1(mg455)* (null #2) (A,C,E), and *sgk-1(ft15) (gf)* (B,D,F). (G) Effect of *daf-16(mu86)* null mutation on the thermotolerance of *sgk-1* null mutants. Raw data and statistics are presented in Tables S2–S4.

In light of our observations on the effects of *sgk-1* mutations on lifespan (Fig. 1), we tested *sgk-1(null)* and *sgk-1(gf)* for their sensitivity to oxidative stress, ultraviolet radiation (UVR), and heat. *akt-1* null mutants were slightly more resistant to hydrogen peroxide than wild-type animals, although this difference was only statistically significant in one of four assays (Fig. 2A,B and Table S2). *akt-1* null mutants were significantly more resistant to UVR and heat than wild-type animals (Fig. 2C–F and Tables S3 and S4). In contrast, both *sgk-1* null mutants were more sensitive to hydrogen peroxide (statistically significant in 2 of 3 trials for each mutant) and UVR (statistically significant in 3 of 3 trials) than wild-type animals (Fig. 2A,C and Tables S3–4), consistent with their short lifespans (Fig. 1B and Table S1). *sgk-1(gf)* did not significantly influence sensitivity to any of the three stressors tested (Fig. 2B,D,F and Tables S3–5).

Both *sgk-1* null mutations enhanced thermotolerance to at least the same extent that an *akt-1* null mutation did (Fig. 2E and Table S4). This result is consistent with a previous report examining thermotolerance of the *sgk-1(ok538)* null mutant (Hertweck *et al*., 2004). Taken together with our observation that the *sgk-1(gf)* mutation extends lifespan (Fig. 1 and Table S1), this enhanced thermotolerance phenotype of *sgk-1* null mutants strengthens the argument that the short-lived phenotype of *sgk-1* null mutants is not simply a consequence of frailty secondary to developmental abnormalities. In contrast to AKT-1, which promotes general sensitivity to environmental stress, SGK-1 is protective against oxidative stress and UVR but enhances sensitivity to heat.

As the thermotolerance of *sgk-1(ok538)* is thought to require DAF-16/FoxO (Hertweck *et al*., 2004), we tested the effect of a *daf-16* null mutation on the thermotolerance of both *sgk-1* null mutants. Surprisingly, *daf-16* null mutation did not significantly influence the thermotolerance of either *sgk-1* null mutant (Fig. 2G and Table S4). Therefore, our results suggest that SGK-1 promotes sensitivity to heat in a DAF-16/FoxO-independent manner.

### Effects of *sgk-1* mutations on DAF-16A::GFP subcellular localization

**Fig. 3:**
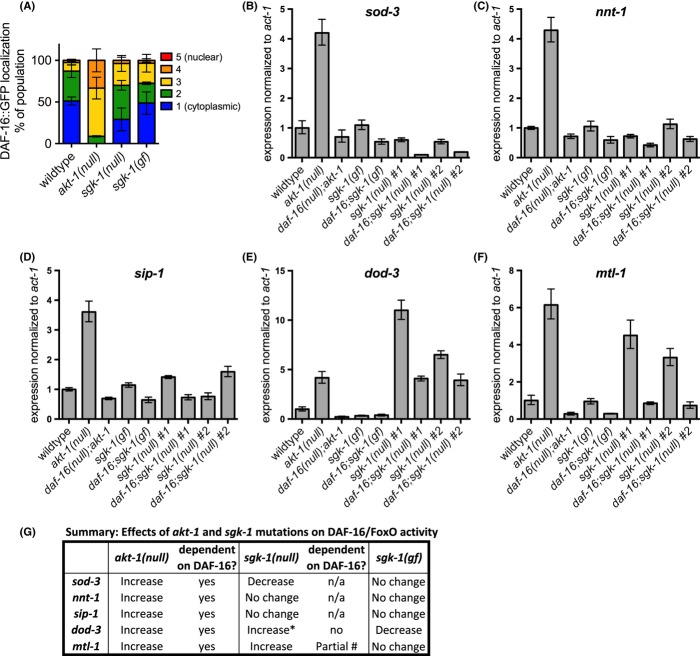
Effects of *sgk-1* mutations on DAF-16A::GFP subcellular localization and DAF-16/FoxO target gene expression. (A) Subcellular localization of DAF-16A::GFP in *akt-1* and *sgk-1* mutants. Nuclear localization is increased by *akt-1(mg306)* null mutation (two-way anova, *F* = 14.47, *P* < 0.0001), but not by *sgk-1(ok538)* null mutation (*F* = 1.825, *P* = 0.1733) or by *sgk-1(ft15)* gain-of-function mutation (*F* = 0.869, *P* = 0.5037). Error bars represent SEM for 3 cohorts of 20–30 animals per genotype imaged separately. All animals also harbored the *daf-16(mu86)* null allele, so no endogenous DAF-16/FoxO is present. Representative images are shown in Figure S2. Raw data and statistics are presented in Table S5. (B,F) Representative experiments measuring *sod-3* (B), *nnt-1* (C), *sip-1* (D*), dod-3* (E), and *mtl-1* (F) transcript levels using quantitative RT–PCR on total RNA isolated from young adult animals. Values are normalized to expression levels in wild-type animals. Columns represent mean ± SEM of three technical replicates. Raw data and statistics for biological replicates are summarized in Table S6. (G) Summary of statistically significant gene expression changes (*P* < 0.05; Table S6; unpaired two-tailed *t*-test with Welch's correction) in *akt-1* and *sgk-1* mutants and their dependence on DAF-16/FoxO. The asterisk indicates that *dod-3* expression was increased significantly in eight of twelve trials. The number sign indicates that *daf-16(null)* significantly reduced expression of *dod-3* and *mtl-1* in *sgk-1(null)* mutants in five of six trials. See Table S6 for details.

As our lifespan data are consistent with a model in which SGK-1 promotes longevity by activating DAF-16/FoxO, we sought to determine the influence of *sgk-1* mutations on the subcellular localization of DAF-16/FoxO. Sgk promotes the nuclear export and cytoplasmic sequestration of FoxO in mammalian cells (Brunet *et al*., 2001); however, based on conflicting reports in the literature (Hertweck *et al*., 2004; Kwon *et al*., 2010), the role of *C. elegans* SGK-1 in regulating DAF-16/FoxO localization remains unclear. We constructed *sgk-1(null)* and *sgk-1(gf)* strains expressing a functional DAF-16A::GFP fusion protein as the sole source of DAF-16/FoxO in the animal and determined DAF-16A::GFP subcellular localization in young adult animals raised in the same conditions used for lifespan assays (Fig. 3A and Table S5). Under these conditions, *akt-1* null mutation promoted the translocation of DAF-16A::GFP from the cytoplasm to the nucleus, as previously shown (Zhang *et al*., 2008; Alam *et al*., 2010; Dumas *et al*., 2010). Neither the *sgk-1(ok538)* null mutation nor *sgk-1(gf)* had a significant influence on the nucleocytoplasmic distribution of DAF-16A::GFP. These data suggest that unlike AKT-1, SGK-1 does not control DAF-16/FoxO activity *in vivo* by regulating its subcellular localization.

### Effects of *sgk-1* mutations on DAF-16/FoxO target gene expression

The dependence of *sgk-1(gf)* lifespan extension on *daf-16/FoxO* (Fig. 1C) suggests that SGK-1 may increase lifespan by activating DAF-16/FoxO, even in the absence of a significant effect on DAF-16A::GFP localization (Fig. 3A). Therefore, we quantified the expression of five DAF-16/FoxO target genes (Murphy *et al*., 2003; Oh *et al*., 2006; Alam *et al*., 2010; Dumas *et al*., 2010; Kwon *et al*., 2010) in young adult *sgk-1(null)* and *sgk-1(gf)* animals (Fig. 3B–F and Table S6).

As expected for bona fide DAF-16/FoxO target genes, the expression of all five of these genes is increased in a DAF-16/FoxO-dependent manner in the context of *akt-1* null mutation (Fig. 3B–F and Table S6) (Alam *et al*., 2010; Dumas *et al*., 2010). In contrast, *sgk-1* mutations had varying influences on the expression of these five genes. *sod-3* expression was not influenced by *sgk-1(gf)* but was reduced in *sgk-1* null mutants (Fig. 3B and Table S6). Thus, null mutations in *sgk-1* and *akt-1* have opposite effects on *sod-3* expression. Neither *sgk-1* null mutation nor *sgk-1(gf)* reproducibly influenced the expression of *nnt-1* and *sip-1* (Fig. 3C,D and Table S6). Expression of both *dod-3* and *mtl-1* was elevated in a DAF-16/FoxO-dependent manner in the context of *sgk-1* null mutation in five of six trials (Fig. 3E,F and Table S6), suggesting that SGK-1 and AKT-1 may act similarly to regulate these two DAF-16/FoxO target genes.

In aggregate, these data indicate that AKT-1 and SGK-1 control DAF-16/FoxO target gene expression through distinct mechanisms. The heterogeneity of the influence of *sgk-1* mutations on DAF-16/FoxO target gene expression suggests that the molecular basis for SGK-1 regulation of DAF-16/FoxO activity is significantly more complex than has been appreciated.

## Discussion

Akt/PKB inhibits FoxO transcription factors via a well-established and evolutionarily conserved mechanism involving phosphorylation of FoxO at three sites that lie within conserved RxRxxS/T motifs (Manning & Cantley, 2007; Franke, 2008). Based on both its similarity in primary structure (Webster *et al*., 1993) and substrate specificity (Kobayashi & Cohen, 1999) to Akt/PKB as well as data from mammalian cell culture (Liu *et al*., 2000; Brunet *et al*., 2001) and *C. elegans* (Hertweck *et al*., 2004), Sgk is also thought to inhibit FoxO by promoting its phosphorylation at RxRxxS/T motifs. Our data challenge this model of FoxO regulation by Sgk and support the notion that in *C. elegans,* Akt/PKB and Sgk regulate FoxO activity in fundamentally different ways.

Our conclusions are at odds with those of the only study in the literature that has focused on Sgk action in *C. elegans* lifespan control and FoxO regulation (Hertweck *et al*., 2004). This study showed that *sgk-1* RNAi extends lifespan in a DAF-16-/FoxO-dependent manner. One possible explanation for this discrepancy is that the *E. coli* strain used for RNAi (the HB101-related strain HT115) is different from the standard strain used for growth and maintenance of *C. elegans* (the *E. coli* B-related OP50) that we used in our experiments. Indeed, wild-type *C. elegans* grown on HT115 live nearly 20% longer than wild-type animals grown on OP50 (Maier *et al*., 2010). However, we have shown that *sgk-1(null)* and *sgk-1(gf)* animals are respectively short-lived and long-lived when cultured on *E. coli* OP50, HT115, or HB101 (Fig. 1 and Table S1), indicating that the lifespan phenotypes of *sgk-1(null)* and *sgk-1(gf)* are not significantly influenced by differences between OP50 and HT115/HB101 *per se*.

We did confirm the previously reported finding that *sgk-1* null mutant animals are thermotolerant compared with wild-type animals (Hertweck *et al*., 2004). This suggests that *sgk-1* null mutant animals are not short-lived due to general frailty or sickness, as such animals would be expected to be generally hypersensitive to environmental stresses. Intriguingly, *daf-16/FoxO* was not required for thermotolerance in *sgk-1(null)* animals, suggesting that although AKT-1 and SGK-1 both promote thermosensitivity, they likely do so through distinct mechanisms. Our results dissociate thermotolerance from longevity and suggest that divergent molecular pathways act downstream of SGK-1 to influence lifespan and responses to increased ambient temperature.

Our results also contrast with a detailed analysis of mammalian FoxO3 regulation demonstrating that both Sgk and Akt/PKB can inhibit FoxO3 activity in cell culture by promoting the phosphorylation of all three conserved sites that lie within RxRxxS/T motifs (Brunet *et al*., 2001). This discrepancy may be due in part to differences in experimental context; these experiments were performed in cell culture, where growth factors are frequently added in excess of physiologic concentrations, and overexpressed proteins may exhibit activities that are not discernible when they are expressed at endogenous levels. The effect of Sgk knockdown or deletion on the activity of endogenous FoxO transcription factors has not been investigated in mammals. Although it is conceivable that Sgk regulates FoxO activity through distinct mechanisms in mammals and *C. elegans*, this is unlikely in light of the conservation of mechanisms of FoxO regulation by insulin-like growth factor signaling pathways (Kenyon, 2010).

Although the increased lifespan phenotypes caused by *akt-1* null mutation and the *sgk-1(gf)* both require *daf-16/FoxO* (Fig. 1C and Table S1), the expression of DAF-16/FoxO target genes was influenced by these two mutations in starkly discordant ways (Fig. 3B–F). Whereas the expression of five DAF-16/FoxO target genes is induced in a DAF-16/FoxO-dependent manner in *akt-1* null mutants, *sgk-1(null)* and *sgk-1(gf)* mutations had distinct and varying influences on the expression of specific DAF-16/FoxO target genes. This difference is likely a reflection of underlying differences in the molecular basis for DAF-16/FoxO regulation by AKT-1 and SGK-1.

These observations suggest that the underlying mechanisms of lifespan control by AKT-1 and SGK-1 are fundamentally different. In contrast to AKT-1, which inhibits DAF-16/FoxO by promoting its nuclear export and cytoplasmic retention (Hertweck *et al*., 2004; Zhang *et al*., 2008; Alam *et al*., 2010; Dumas *et al*., 2010), SGK-1 may promote longevity by regulating other proteins that functionally and/or physically interact with DAF-16/FoxO, such as SKN-1 (Tullet *et al*., 2008), HSF-1 (Hsu *et al*., 2003), or HCF-1 (Li *et al*., 2008). In this regard, DAF-16/FoxO may play a permissive role in lifespan control by SGK-1 without being directly regulated by SGK-1. Alternatively, SGK-1 may directly regulate DAF-16/FoxO activity in a small number of cells, which in turn could control lifespan by influencing other cells in a DAF-16/FoxO-independent manner.

In summary, we have shown that the AGC kinase family members Akt/PKB and Sgk control *C. elegans* lifespan and stress resistance in fundamentally different ways, and they likely influence FoxO transcription factor activity through distinct mechanisms *in vivo*. Our findings challenge existing paradigms of FoxO regulation by Sgk and should engender a reassessment of the role of Sgk in FoxO transcription factor regulation.

## Experimental procedures

### Strains and reagents

The following strains were used: N2 Bristol (wild-type), *sgk-1(ft15)* (Jones *et al*., 2009), *akt-1(mg306)* (Hu *et al*., 2006), *sgk-1(ok538)* (Hertweck *et al*., 2004), *sgk-1(mg455)* (Soukas *et al*., 2009), *daf-16(mu86)* (Lin *et al*., 1997), *hcf-1(pk924)* (Li *et al*., 2008), and TJ356 (*zIs356*) (Henderson & Johnson, 2001). Because *sgk-1(ft15)* was isolated after mutagenesis of animals harboring the linked *akt-2(tm812)* mutation (Jones *et al*., 2009), we confirmed the absence of *akt-2(tm812)* prior to further analysis. Throughout the manuscript, *sgk-1(ft15)* is referred to as ‘*sgk-1(gf)’*, *akt-1(mg306)* as ‘*akt-1(null)’*, *sgk-1(ok538)* as ‘*sgk-1(null)* #1’, and *sgk-1(mg455)* as ‘*sgk-1(null)* #2’. *sgk-1* mutant strains were outcrossed with N2 at least seven times prior to phenotypic analysis. Wild-type siblings of *sgk-1*(*ft15*) from the seventh outcross with N2 Bristol were used as controls for phenotypic comparison to *sgk-1(ft15)*. This sibling is labeled ‘wild-type’ in all figures, in contrast to ‘N2 wild-type’. Double and triple mutants were generated using standard genetic techniques. For maintenance and all assays, animals were grown in Percival I-30NL or I-36NL incubators (Percival Scientific, Inc., Perry, IA, USA).

### Lifespan assays

Lifespan assays were performed at 20°C as described (Alam *et al*., 2010; Dumas *et al*., 2010). Briefly, animals were treated with alkaline hypochlorite and grown for at least three generations at 15°C. A synchronized egg lay was then performed to yield animals for the lifespan assay. These were grown at 20°C until the L4 larval stage, at which time they were picked to separate plates and grown until they were day 2 adults. They were then transferred to NGM plates (10–15 animals per plate) containing 25 μg/mL (100 μM) 5-fluoro-2′-deoxyuridine (FUDR; Sigma-Aldrich, St. Louis, MO, USA) and 10 μg/mL nystatin (Sigma-Aldrich) and seeded with 20× concentrated OP50. Animals were incubated at 20°C and scored every 1–2 days. Animals that were not moving, did not respond to prodding, and did not exhibit pharyngeal pumping were scored as dead and removed. Animals that died due to desiccation on the side of the plate, a compromise in vulval integrity, or bagging were censored. Statistical significance was assessed using the standard chi-square-based log-rank test in GRAPHPAD PRISM (GraphPad Software, La Jolla, CA, USA).

### Dauer assays

Dauer assays were performed at 25° or 27°C as previously described (Hu *et al*., 2006). Briefly, animals were synchronized in a 4- to 6-h egg lay and grown at 25° or 27°C on NGM plates. Dauers were scored when wild-type animals were gravid adults and *daf-2(e1368)* or *akt-1(mg306)* mutant animals were arrested as dauers (approximately 60–84 h after egg lay). *sgk-1* null mutant animals were plated twelve hours prior to other strains to compensate for developmental delay. Plates were observed for two additional days after initial scoring to account for possible dauer arrest in animals with severe developmental delay.

### Stress resistance assays

Animals were grown at 20°C for 48 hours after a 4- to 6-h egg lay until most animals were L4 larvae. *sgk-1(null)* animals were grown starting 12 h earlier than other strains for L4 synchronization due to developmental delay (Hertweck *et al*., 2004; Jones *et al*., 2009; Soukas *et al*., 2009). Young adults, L3 larvae, and males were removed by suction. Cohorts were sufficiently large to allow for thermotolerance, oxidative stress, and UV assays to be performed in parallel. All assays were performed in triplicate.

For oxidative stress assays, L4 larvae were transferred to fresh seeded NGM plates, grown for an additional 18 h, washed two or three times with M9 buffer, and diluted to a concentration of ∼ 50 animals mL^−1^ of M9. 0.5 mL of animals was dispensed to Eppendorf tubes and rocked for ∼ 20 min to allow animals to digest *E. coli*. Four tubes were used per genotype per concentration of H_2_O_2_. 0.5 mL of H_2_O_2_ dissolved in M9 was then added to each tube to the final concentration, followed by rocking for 2 h protected from light. The H_2_O_2_ solution was then removed, and the animals were washed with M9. Animals were then pipetted back onto fresh NGM plates and scored after an 18-h recovery period at 20°C. Two-way anova was conducted using GraphPad Prism, with survival of animals on each plate as the dependent variable and H_2_O_2_ dose and genotype as independent variables.

UV stress assays were performed as described (Wolff *et al*., 2006). Briefly, animals were transferred to plates containing 25 μg mL^−1^ FUDR on day 1 of adulthood. After four days, they were transferred to plates lacking bacteria and irradiated with 1200 J m^−2^ UV-C using a Stratalinker 2400 UV crosslinker (Stratagene, La Jolla, CA, USA). They were then transferred onto NGM plates with food and scored daily for survival. Statistical significance was assessed using the standard chi-square-based log-rank test.

Thermotolerance assays were performed essentially as described (Kwon *et al*., 2010). Briefly, L4 larvae were transferred to fresh seeded NGM plates (∼20 per plate) and then grown for an additional 18 h prior to shifting them to an incubator set at 35°C. Four plates were used per genotype per time point. At each time point, plates to be scored were removed and incubated further for 18 h at 20°C, after which living and dead animals were scored. Two-way anova was conducted using GRAPHPAD PRISM, with survival of animals on each plate as the dependent variable and time at 35°C and genotype as independent variables.

### DAF-16A::GFP localization assays

Animals were mounted onto slides in M9 with 10 mm sodium azide. Approximately ten young adults were picked to each slide, and the anterior segment of each animal was imaged within five minutes after mounting. Images were scored according to the criteria shown in Figure S1. Both imaging and scoring were performed in a blinded fashion. Two-way anova was used to assess statistical significance in GRAPHPAD PRISM.

### Quantitative RT–PCR

Animals from a 4.5-h egg lay were grown at 20°C for 48 h until most animals were L4 larvae. *sgk-1(null)* animals were grown starting 12 h earlier than other strains for L4 synchronization due to developmental delay (Hertweck *et al*., 2004; Jones *et al*., 2009; Soukas *et al*., 2009). Young adults and L3 larvae were removed by suction, and the remaining animals were grown for an additional 12 h. Total RNA was isolated from 600–1000 young adults per strain per biological replicate using TRIzol (Invitrogen, Carlsbad, CA, USA) and purified using an RNeasy Kit (QIAGEN Inc., Valencia, CA, USA). cDNA was synthesized using a Superscript III Reverse Transcriptase Kit (Invitrogen). SYBR Green (Applied Biosystems, Warrington, UK) Real-Time PCR was then performed using primers corresponding to the DAF-16/FoxO target genes *sod-3, nnt-1, sip-1, dod-3,* and *mtl-1*. *act-1* was used as an internal control. Quantitative PCR primer sequences are listed in Table S7. Statistical analysis was performed in GraphPad Prism by unpaired two-tailed *t*-test with Welch's correction.

## Supporting Information

**Fig. S1** Criteria for scoring of DAF-16A::GFP subcellular localization.

**Fig. S2** Representative photographs of wild-type, *sgk-1(null), sgk-1(gf)*, and *akt-1(null)* animals expressing DAF-16A::GFP.

**Fig. S3** Photograph of arrested *sgk-1(null)* nondauer larva.

**Table S1** Lifespan data and statistics relevant to Fig. 1.

**Table S2** H_2_O_2_ survival data and statistics for each replicate of Fig. 2A,B.

**Table S3** UV survival data and statistics for each replicate of Fig. 2C,D.

**Table S4** Thermotolerance data and statistics for each replicate of Fig. 2E–G.

**Table S5** Raw DAF-16A::GFP subcellular localization data and statistics for Fig. 3A.

**Table S6** qPCR data and statistics for each replicate of Fig. 3B–F.

**Table S7** qPCR primers for Fig. 3B–F.
