# Supplementary material for: Effects of Caenorhabditis elegans sgk-1 mutations on lifespan, stress resistance, and DAF-16/FoxO regulation
Source: Aging Cell. 2013 Jul 19;12(5):932–40. doi: 10.1111/acel.12120 (PMC3824081; doi:10.1111/acel.12120)
Supplement: Supplementary file 1 [file acel0012-0932-SD1.docx]

**Figure S1**

**Figure S1. Criteria for blind scoring of DAF-16::GFP subcellular localization.** *daf-16(mu86)* mutant animals carrying high-copy DAF-16::GFP (TJ356, Henderson and Johnson, 2001) and *sgk-1* or *akt-1* alleles (see Figure S3) were grown at 20°C and picked to slides as young adults. The anterior of each animal was imaged and scored blindly and classified into one of five groups by the criteria shown.

**Figure S2**

**Figure S2. Example photographs of wildtype, *sgk-1(null), sgk-1(gf)*, and *akt-1(null)* animals expressing DAF-16::GFP.** *daf-16(mu86)*;DAF-16::GFP (TJ356, Henderson and Johnson, 2001) were built with wildtype (A), *akt-1(mg306)* null (B), *sgk-1(ft15)* gain-of-function (C), *sgk-1(ok538)* null (D) alleles, and imaged and scored as described in Fig. S1. *sgk-1(ok538)* null animals showed autofluorescent granules (inset) in their intestines that were too small to be nuclei and were present in non-GFP animals, as early as the L2 larval stage (E), and thus were not scored as nuclei in DAF-16::GFP localization assays (F).

**Figure S3**

**Figure S3. Example photograph of the pharynx of a arrested *sgk-1(null)* larva at 27°C (lower panel).** For comparison, a remodeled pharynx of a *daf-2(e1368)* dauer is shown in the upper panel.

**Table S1**

**Table S1.** **Life span data and statistics for (A) each replicate of Fig. 1**, (B) replicates using N2 wildtype as a control, and (C) replicates feeding HB101 or HT115 bacteria. All assays were conducted at 20°C. We used GraphPad Prism (GraphPad Software; La Jolla, CA, USA) to perform statistical analysis, including the log-rank (Mantel-Cox) test. Data used in Figures 1B-1E are labeled as “* shown figure.”

**Table S2**

**Table S2. H_2_O_2_ survival data and statistics for each replicate of Fig. 2A and 2D**. Young adult animals were subjected to H_2_O_2_ treatment in M9 for 2 hours in Eppendorf tubes, allowed to recover for 18 hours, and then scored for survival. Animal counts listed for each genotype are split among 5 treatments ranging from 200 to 400 μM and between 4 tubes per genotype per treatment. Survival for each tube was calculated, allowing mean and median lethal dose to be calculated. Pairing by H_2_O_2_ concentration was used to conduct a Student’s t-test in GraphPad Prism. Data used in Figures 2A and 2D are labeled as ‘*.’

**Table S3**

**Table S3. UV survival data and statistics for each replicate of Fig. 2B and 2E.** Synchronized animals were maintained on FUDR plates until they were 4 days old, transferred to plates without food and exposed to ultraviolet radiation (1200 J/m2 UV-C) using a Stratalinker UV Crosslinker. Survival was scored daily for each animal from time of UV exposure until death, similar to life span assays. We used GraphPad Prism to perform statistical analysis, including the log-rank (Mantel-Cox) test. Data used in Figures 2B and 2E are labeled as ‘*.’

**Table S4**

**Table S4. Thermotolerance data and statistics for each replicate of Fig. 2C, 2F, and 2G.** Young adult animals were subjected to 35°C for 3 to 6 hours on NGM plates, allowed to recover for 18 hours, and then scored for survival. Animal counts listed for each genotype are split among 6 or 7 time points and between 4 plates per genotype per time point. Survival for each plate was calculated, allowing mean and median lethal time exposure to be calculated. Pairing by time point was used to conduct a Student’s t-test in GraphPad Prism. Data used in Figures 2C and 2F are labeled as ‘*.’

**Table S5**

**Table S5. DAF-16A::GFP subcellular localization data for Figure 3A.** *daf-16(mu86)* mutant animals carrying a DAF-16A::GFP (TJ356, Henderson and Johnson, 2001) and *sgk-1* or *akt-1* alleles were grown, imaged, and scored as described in Figure S2.

**Table S6**

**Table S6**. **qPCR data and statistics for each replicate of Figure 3B-3F.** Five DAF-16/FoxO targets were each measured in six independent biological replicates, although not all genotypes were included in every replicate. All animals listed in the same column were grown and harvested at the same time under identical conditions. Three technical replicate measurements were performed per target/genotype/biological replicate and compared to three technical replicate measurements of *act-1*. All data is normalized to wildtype within the same cohort. An unpaired two-tailed t-test with Welch’s correction (P < 0.05) was performed to make relevant comparisons, and statistically significant changes are bolded. Note that *sgk-1* null mutant changes in *dod-3* and *mtl-1* gene expression did not always reach statistical significance due to high variability.

**Table S7**

**Table S7. qPCR primers for Figures 3A-3E.**
